
JOURNAL INFORMATION
==============================
NLM Title Abbreviation: Eur J Gen Pract
Journal ID: Eur J Gen Pract
NLM Journal ID: 9513566

The European Journal of General Practice

ISSN: 1381-4788
EISSN: 1751-1402
Publisher: Taylor & Francis

ARTICLE INFORMATION
==============================
PMCID: PMC10885746
DOI: 10.1080/13814788.2024.2312410
Article ID: 2312410
Article version: 1
Article version: Version of Record
Subjects: Abstract, Abstracts

Innovative Technologies and Methods in General Practice: Selected Abstracts from the 97th EGPRN Meeting, Prague, Czech Republic, 12–15 October 2023

All abstracts of the conference can be found at the EGPRN website https://www.egprn.org/page/conference-abstracts

Abstracts

Innovative Technologies and Methods in General Practice

Electronic publication date: 2024 Feb 22
Publication date: 2024
Volume: 30
Issue: 1
Electronic Location ID: 2312410
Copyright: © 2024 The Author(s). Published by Informa UK Limited, trading as Taylor & Francis Group.
Copyright year: 2024
Copyright holder: The Author(s)
License: This is an Open Access article distributed under the terms of the Creative Commons Attribution License (http://creativecommons.org/licenses/by/4.0/), which permits unrestricted use, distribution, and reproduction in any medium, provided the original work is properly cited. The terms on which this article has been published allow the posting of the Accepted Manuscript in a repository by the author(s) or with their consent.
License URL: https://creativecommons.org/licenses/by/4.0/

Figure count: 0
Table count: 0
Page count: 10

==============================


Introduction to the conference theme: ‘Innovative technologies and methods in general practice’

Subjects: Abstract, Keynote Lectures

New technology in clinical practice: Will it help the general practitioner?

de Wit Niek
University Medical Center Utrecht , Utrecht , The Netherlands
CONTACT Niek de Wit n.j.dewit@umcutrecht.nl University Medical Center Utrecht, Utrecht, The Netherlands

Innovative methods in the development of trustworthy clinical practice guidelines

Klugar Miroslav
Department of EBM and Assessment of the Healthcare Quality, Institute of Health Information and Statistics of the Czech Republic , Prague , Czech Republic
CONTACT Miroslav Klugar miloslav.klugar@uzis.czDepartment of EBM and Assessment of the Healthcare Quality, Institute of Health Information and Statistics of the Czech RepublicPrague, Czech Republic

Subjects: Abstract, Poster Prize Winner

Does personalised advice about cervical and breast cancer screening effect women’s intention to be screened?

Ponzel Nataliia
Kolesnyk Pavlo
Uzhhorod National University , Uzhhorod , Ukraine
CONTACT Nataliia Ponzel natalia.ponzel@uzhnu.edu.ua Uzhhorod National University, Uzhhorod, Ukraine
Keywords: Ukrainian ınternally displaced women, screening, breast cancer, cervical cancer, general practice/family medicine, primary care

Subjects: Abstract, SELECTED ABSTRACTS Themed topics

Using information technology to facilitate research in a practice-based research network - the story of PraksisNett

Fossum Guro Haugen
Halvorsen Peder
University of Oslo , Oslo , Norway
CONTACT Guro Haugen Fossum g.h.fossum@medisin.uio.noUniversity of Oslo, 0318Oslo, Norway
Keywords: Clinical interventions, family practice, health services research, quality development, primary care, general practice, practice-based research networks

Conceptual overview of telemedicine in primary care: A grounded theory perspective

Thulesius Hans
Arvidsson Eva
Sanden Ulrika
Wilkens Jens
Ekman Björn
R&D Kronoberg, Clinical Sciences , Växjö , Sweden
CONTACT Hans Thulesius hansthulesius@gmail.comR&D Kronoberg, Clinical Sciences, Växjö, Sweden
Keywords: Telemedicine, primary care, digital text contacts, grounded theory

Identifying available addictive disorder screening tests feasible in primary care: A systematic review

Pautrat Maxime
Maison de Santé Pluridisciplinaire, Médecine Générale , Lıgueıl , France
CONTACT Maxime Pautrat lamibaryton@hotmail.fr Maison de Santé Pluridisciplinaire, Médecine Générale, Lıgueıl, France
Keywords: Feasibility, substance use disorders, screening, primary care

Intention to treat and per protocol analyses showed different results in a diagnostic cluster randomised controlled trial evaluating faecal calprotectin in primary care

Ansems Sophie
Berger Marjolein
Van Rheenen Patrick
Vermeulen Karin
De Boer Michiel
Holtman Gea
University Medical Center Groningen , Groningen , The Netherlands
CONTACT Sophie Ansems s.m.ansems@umcg.nl University Medical Center Groningen, Groningen, The Netherlands.

How to implement a physical activity programme in primary care among cancer survivors. Barriers and facilitators from the perspective of the gp and practice nurse

Huizinga Famke
Westerink Nico-Derk Lodewijk
Berendsen Annette J.
Walenkamp Annemiek M.e.
Berger Marjolein Y.
Brandenbarg Daan
University Medical Center Groningen , Eelde , The Netherlands
CONTACT Famke Huizinga f.huizinga@umcg.nl University Medical Center Groningen, Eelde, Netherlands

Evaluation of the effectiveness of quality committees in primary healthcare in Tajikistan

Dastan Ilker
Dzhonova Bunafsha
Miraliev Salohiddin
Makhmudova Parvina
Bohlmann Natascha
Olimova Lola
World Health Organization , Dushanbe , Tajikistan
CONTACT Ilker Dastan dastani@who.int World Health Organization, Dushanbe, Tajikistan
Keywords: Quality committee, effectiveness, primary health care, maternal and child health, tajikistan

Digital self-triage: How compliant is the patient?

Pairon Anthony
Verhoeven Veronique
Philips Hilde
University of Antwerp , Antwerp , Belgium
CONTACT Anthony Pairon anthony.pairon@student.uantwerpen.be University of Antwerp, Antwerp, Belgium
Keywords: Triage, symptom checker, self-triage, digital, e-health, m-health, care-seeking behaviour, compliance

Subjects: FREESTANDING Healthcare delivery

The inverse care law as applied to general practice clinics in the irish midwest: Examining effects through simulated practice closure

O'Callaghan Michael
Harbour Eric
Stanley Fintan
Liam Glynn Irish College of General Practitioners , Dublin , Ireland
CONTACT Michael O'Callaghan mike.ocallaghan@icgp.ie Liam Glynn Irish College of General Practitioners, Dublin, Ireland

Subjects: Abstract, Clinical Topics

Which patients participate in diabetes self-management education (DSME), what are the reasons for non-participation, and how do participants rate DSME training? Data from a nationwide survey

Weise Solveig
Baumert Jens
Heidemann Christin
Du Yong
Frese Thomas
Heise Marcus
Institute of General Practice and Family Medicine, Medical Faculty, Martin-Luther-University Halle-Wittenberg , Halle (Saale) , Germany
CONTACT Solveig Weise solveigweise@posteo.de Institute of General Practice and Family Medicine, Medical Faculty, Martin-Luther-University Halle-Wittenberg, Halle (Saale), Germany
Keywords: Diabetes self-management education, population-based study, cross-sectional study, reasons to decline patient education

How often do we measure blood pressure in the office? Results of the Hungarian hypertension registry

Nemcsik János
Takács Johanna
Farsang Csaba
Simon Attila
Páll Dénes
Torzsa Péter
Dolgos Szilveszter
Koller Akos
Járai Zoltán
Department of Family Medicine, Semmelweis University , Budapest , Hungary
CONTACT János Nemcsik janos.nemcsik@gmail.com Semmelweis University, Department of Family Medicine, Budapest, Hungary

Up-rise ability predicted hip fractures and mortality in 295 women 70 years and older – a 20 year follow-up controlled intervention

Albertsson Daniel
Alvunger Lisa
Lindgren Anna
Mölstad Ulrica
Petrazzuoli Ferdinando
Segernäs Anna
Sjölander Moses
Thulesius Hans
Wanby Pär
Eggertsen Robert
Research and Development Unit, School of Public Health and Community Medicine, University of Gothenburg , Vaxjo , Sweden
CONTACT Daniel Albertsson daniel.albertsson@kronoberg.se School of Public Health and Community Medicine, University of Gothenburg. Research and Development Unit, Vaxjo, Sweden
Keywords: Hip fracture, uprise, women, follow-up intervention study, population-based cohort, primary care

Prevention needs in adolescents: What is their emotional problem all about?

González Inés Alonso
Docampo Macarena Chacón
Pastoriza Sara Rodríguez
Mariñas David Liñares
Diego Lorena Comesaña
Fontán Ana Clavería
Instituto Investigación Sanitaria Galicia Sur. Grupo I-Saúde , Vıgo , Spain
CONTACT Macarena Chacón Docampo macachd@gmail.com Instituto Investigación Sanitaria Galicia Sur. Grupo I-Saúde, Vıgo, Spain
Keywords: Adolescents, emotional and behavioural disorders, community care

Subjects: Abstract, Covid

Degree of self-perceived disability and impact on their quality of life in primary care healthcare workers with long COVID-19: EPICOVID-AP21 study

González-Lama Jesús
Villatoro Jaime Monserrat
Ranchal-Sánchez Antonio
Carmona-Casado Ana Belén
Pérula-Detorres Luis Ángel
Castro-Jiménez Rafael Ángel
Jiménez-García Celia
Ramírez-Baena Miguel
Romero-Rodríguez Esperanza
Maimonides Biomedical Research Institute of Cordoba (IMIBIC) , Cabra , Spain
CONTACT Jesús González-Lama jesus.gonzalez.sspa@juntadeandalucia.es Maimonides Biomedical Research Institute of Cordoba (IMIBIC), Cabra, Spain

Delphi study on primary healthcare indicators for COVID-19 pandemic across 30 European countries: Eurodata study, preliminary results

Bravo Raquel Gomez
Blanco Sara Ares
Larrondo Ileana Gefaell
Rio Lourdes Ramos Del
Petrazzuoli Ferdinando
Serafini Alice
Petek Davorina
Knezevic Snezana
Assenova Radost
Frese Thomas
Lingner Heidrun
Adler Limon
Vinker Shlomo
Neves Ana Luisa
Heleno Bruno
Brutskaya-Stempkovskaya Elena
Vaes Bert
Seifert Bohumil
Pencheri Maria
Bayen Sabine
Sentker Theresa
Torzsa Peter
Fitzgerald Louise
Sattler Martin
Peña Maryher Delphin
Nessler Katarzyna
Busneag Carmen
Kvitting Anna
Kırkoç Erva
Korkmaz Büsra Çimen
Ільков Оксана
Penakacherla Nagu
Gjorgjievski Dragan
Kirkovski Aleksandar
Bakola Maria
Domeyer Philip
Bensemmane Sherihane
Perjés Ábel
Hoffmann Kathryn
Hanževački Miroslav
Kostić Milena
Petricek Goranka
Jandrić-Kočić Marijana
Mortsiefer Achim
Burgers Jako
Buitrago Alejandro Gimenez
Moran Pedro Jose Saurin
Clavero Marina Guisado
Peña Maria Pilar Astier
Eurodata Collaborative Group
Spanish Society of Family and Community Medicine semFYC , Madrid , Spain
CONTACT Sara Ares Blanco sararesb@yahoo.es Spanish Society of Family and Community Medicine semFYC, Madrid, Spain
Keywords: Covıd-19, indicators, primary healthcare, public health

Knowledge, attitudes, and perceptions towards vaccination against COVID-19 in a Greek Island community

Psaraki Evgenia
Bakola Maria
Kitsou Konstantina Soultana
Charalambous George
Jelastopulu Eleni
School of Medicine, University of Patras , Rio Patras , Greece
CONTACT Eleni Jelastopulu jelasto@upatras.gr University of Patras, School of Medicine, Rio Patras, Greece
Keywords: Cross-sectional study, covıd-19, vaccination status, vaccination reluctance, Greece

Side effects of the COVID-19 vaccine in the vaccinated population of vlora district; evidence of the efficacy and safety of the vaccines applied

Zahaj Majlinda
Saliaj Aurela
Prifti Vasilika
Nikaj Sonila
Allushi Evis
Selfo Denada
Kicaj Emirjona
Brokaj Stiliana
Malaj Krenar
University Ismail Qemali, Vlore Albania , Vlore , Albania
CONTACT Vasilika Prifti vasilika.prifti@yahoo.com University Ismail Qemali, Vlore Albania, Vlore, Albania
Keywords: COVID-19 vaccines, booster dose, side effects, challenges

Burnout among Catalan family doctors. Three times cross-sectional study during and after the COVID-19 pandemic

Castellanos Maria Miñana
Marques Maria Teresa Santos E. Silva Caldeira
Martín María Isabel Fernández San
Barragan María Rodríguez
Benaiges Enric Aragonés
Sisó Antoni
Gallisà Josep Basora
Idiap Jordi Gol, Unitat Suport Recerca , Barcelona , Spain
CONTACT Maria Miñana Castellanos mariaminyanacastellanos@gmail.com Idiap Jordi Gol, Unitat Suport Recerca, Barcelona, Spain
Keywords: Burnout, primary healthcare, covıd-19

